# Porcine Reproductive and Respiratory Syndrome Virus Type 1.3 Lena Triggers Conventional Dendritic Cells 1 Activation and T Helper 1 Immune Response Without Infecting Dendritic Cells

**DOI:** 10.3389/fimmu.2018.02299

**Published:** 2018-10-02

**Authors:** Elise Bordet, Fany Blanc, Mathieu Tiret, Elisa Crisci, Edwige Bouguyon, Patricia Renson, Pauline Maisonnasse, Mickael Bourge, Jean-Jacques Leplat, Elisabetta Giuffra, Luc Jouneau, Isabelle Schwartz-Cornil, Olivier Bourry, Nicolas Bertho

**Affiliations:** ^1^Virologie et Immunologie Moléculaire, Institut National de la Recherche Agronomique, Université Paris-Saclay, Jouy-en-Josas, France; ^2^UMR Génétique Animale et Biologie Intégrative, INRA, AgroParisTech, Université Paris-Saclay, Jouy-en-Josas, France; ^3^Department of Population Health and Pathobiology, College of Veterinary Medicine, North Carolina State University, Raleigh, NC, United States; ^4^Virologie et Immunologie Porcines, Agence Nationale de Sécurité Sanitaire, Ploufragan, France; ^5^Université Bretagne Loire, Rennes, France; ^6^Union des Groupements de Producteurs de Viande de Bretagne (UGPVB), Rennes, France; ^7^Institute for Integrative Biology of the Cell (I2BC), CEA, CNRS, Université Paris-Sud, Université Paris-Saclay, Gif-sur-Yvette, France

**Keywords:** PRRSV, Lena, lung, dendritic cells, cDC1, Th1 response, cellular response

## Abstract

Porcine Reproductive and Respiratory Syndrome virus (PRRSV) is an arterivirus responsible for highly contagious infection and huge economic losses in pig industry. Two species, PRRSV-1 and PRRSV-2 are distinguished, PRRSV-1 being more prevalent in Europe. PRRSV-1 can further be divided in subtypes. PRRSV-1.3 such as Lena are more pathogenic than PRRSV-1.1 such as Lelystad or Flanders13. PRRSV-1.3 viruses trigger a higher Th1 response than PRRSV-1.1, although the role of the cellular immune response in PRRSV clearance remains ill defined. The pathogenicity as well as the T cell response inductions may be differentially impacted according to the capacity of the virus strain to infect and/or activate DCs. However, the interactions of PRRSV with *in vivo*-differentiated-DC subtypes such as conventional DC1 (cDC1), cDC2, and monocyte-derived DCs (moDC) have not been thoroughly investigated. Here, DC subpopulations from Lena *in vivo* infected pigs were analyzed for viral genome detection. This experiment demonstrates that cDC1, cDC2, and moDC are not infected *in vivo* by Lena. Analysis of DC cytokines production revealed that cDC1 are clearly activated *in vivo* by Lena. *In vitro* comparison of 3 Europeans strains revealed no infection of the cDC1 and cDC2 and no or little infection of moDC with Lena, whereas the two PRRSV-1.1 strains infect none of the 3 DC subtypes. *In vitro* investigation of T helper polarization and cytokines production demonstrate that Lena induces a higher Th1 polarization and IFNγ secretion than FL13 and LV. Altogether, this work suggests an activation of cDC1 by Lena associated with a Th1 immune response polarization.

## Introduction

The Porcine Reproductive and Respiratory Syndrome virus (PRRSV) is responsible for huge economic losses in pig industry. PRRSV belongs to the *Nidovirales* order, the *Arteriviradae* family, and the *Porartevirus* genus (ICTV 2017 Release). Two different species, PRRSV-1 and PRRSV-2 are now distinguished (1). PRRSV-1 have further been divided into 4 subtypes. PRRSV-1 subtype 1 (PRRSV-1.1) is present in all part of Europe, while PRRSV-1.2, 1.3, and 1.4 are mostly present in Eastern Europe (2). PRRSV-1.3 such as Lena, are more pathogenic than PRRSV-1.1 as Lelystad virus (LV) (3–6). The infection by PRRSV-1.3 is characterized by higher body temperature, more sever clinical signs and lung pathology compared to PRRSV-1.1, whereas viremia and lung viral load are not consistently higher (5, 7).

A lag of several weeks in the clearance of the PRRSV has been observed, mostly attributed to a delay in neutralizing antibodies appearance, although an inhibition of the cellular IFNγ response, less studied, might also be involved [for review see (8, 9)]. It has been reported that virulent PRRSV-1.3 induced a strong early inflammatory response associated with an enhanced adaptive cellular immune response that may participate to their higher pathogenicity (5).

The main cellular targets of PRRSV are macrophages (10). Extracellular sialoadhesin (CD169/Siglec-1) mediates viral internalization via interaction with viral protein GP5/M heterodimer while CD163 receptor plays a role in viral internalization and disassembly interacting with GP2 and GP4 viral proteins (11). In addition to macrophages, other immune cells have been described to be permissive to PRRSV *in vitro*. Cultured blood monocytes, monocyte-derived dendritic cells (moDCs) (12) and bone marrow-derived dendritic cells (bmDC) (13) are susceptible to infection by a large range of PRRSV-1 strains. Nevertheless, *in vitro* differentiation conditions might strongly impact the susceptibility of DC/macrophages to PRRSV (14). In 2013, Frydas et al. showed that virulent PRRSV-1.3 such as Lena were able, *ex vivo*, to infect a broader range of monocytic lineage subpopulations than LV PRRSV-1.1 (15).

Dendritic cells (DCs) and macrophages are mononuclear phagocyte cells (MPC) belonging to the mononuclear phagocyte system (16). DCs are key inducers of adaptive immune responses, they migrate from infected tissues to lymph nodes in order to present antigens and induce the humoral and cellular adaptive immune responses. One running hypothesis is that the differences in pathogenicity of the PRRSV strains might be due to their differential abilities to infect or activate DCs, impacting the inflammatory response as well as the mounting of a protective adaptive immune response. Two studies tentatively tackled the lung DCs infection *in vivo* by PRRSV-1 and 2 respectively (17, 18). However, none of them clearly defined nor distinguished DCs and macrophages, leading to results that cannot be clearly interpreted in terms of DCs/PRRSV interactions. We recently identified porcine respiratory DC and macrophage subpopulations and classified them according to a nomenclature proposed by Guilliams et al. (19, 20). In accordance with knowledge in human and mice, we observed that porcine respiratory DCs presented migratory and naïve T-cell stimulation capacities. Conventional DC1 preferentially inducing a T-helper (Th) 1 response, cDC2 a Th2 response and monocyte-derived DC (moDC) a Th17 response. Moreover moDC produced inflammatory cytokines such as IL1 and IL8, and their proportion increased upon viral infection (21). These populations represent *in vivo* differentiated respiratory DCs and macrophages which can be investigated for their interactions with PRRSV in their natural *in vivo* environment.

In order to explore the role of PRRSV/DCs interactions in the induction of the immune response, we studied the infection of primary lung DCs *in vivo* and *in vitro* as well as the impact of PRRSV infection on DCs functionalities. Highly virulent Lena PRRSV-1.3 was tested *in vivo* and compared *in vitro* with two PRRSV-1.1, namely LV and the newly emerging pathogenic Flanders13 (FL13) (15). We found that primary lung DCs were not infected by any of these strains and that a strong cDC1/Type 1 immune response was activated by Lena, but not by FL13 and LV.

## Materials and methods

### Virus production and titration

The 3 strains of PRRSV used in this study were kindly provided by Dr. Hans Nauwynck, (University of Ghent, Belgium). The highly pathogenic Lena PRRSV-1.3 was used for *in vivo* and *in vitro* infections. Lena has been isolated in Belarus in 2007 from a herd with mortality, reproductive failures and respiratory disorders (22). Lelystad virus was identified in the Netherlands in 1991 (23) and Flanders13 13V091 was isolated in Belgium in 2013 in farms experiencing uncommon long-lasting anorexia, fever and respiratory problems within the first 2 weeks after weaning during enzootic PRRSV infection.

Lena viral stock for *in vivo* experiment was produced using SPF piglets AM. The production was tested negative for PCV2, swine Influenza, *Mycoplasma hyopneumoniae*, classical swine fevers as well as African swine fever. A bacteria culture was also negative. For *in vitro* experiments, Lena, Fl13 and LV stocks were produced using fresh SPF primary alveolar macrophages. Supernatants from infected cells were clarified by centrifugation at 3,300 G, filtered on 0.8 μm. Then 30 ml of supernatant were layered on 4 ml 17% sucrose cushion and centrifuged at 100,000 G for 5 h30 min. The pelleted virus was resuspended in RPMI. Titration of viruses were performed on fresh primary alveolar macrophages using Spearman-Karber TCID50 method according to the OIE manual of diagnostic tests (OIE, “Chapter 2.8.7 Porcine Reproductive and Respiratory Syndrome,” Terr. Man., no. May 2015, 2015).

### *In vivo* infection and tissue collection

For *in vivo* experiment, PRRSV infection was performed at ANSES (Ploufragan, France). The animal experiment was authorized by the French Ministry for Research (authorization no. 2015060113297443) and protocols were approved by the national ethics committee (authorization no. 07/07/15-3). Eight Large White piglets coming from a nucleus herd (free from PRRSV, *Actinobacillus pleuropneumoniae, M. hyopneumoniae)* used as control in another publication (24), were housed in biosecurity level-3 air-filtered animal facilities. Treatments, housing, and husbandry conditions were conformed to the European Union Guidelines (Directive 2010/63/EU on the protection of animals used for scientific purposes). At 10 weeks of age, 4 pigs were inoculated intranasally with Lena (5.10^5^ TCID50/per animal in 2.5 ml per nostril). Ten days post-infection, animals were anesthetized (Zoletil, Virbac, France) and exsanguinated. Broncho-alveolar lavage (BAL) cells were collected using 500 ml of PBS + 2 mM EDTA (PBS/EDTA) for each lung. After BAL procedure, peripheral parenchymal tissue from diaphragmatic lobes (PAR) were sampled, minced and incubated in complete RPMI consisting of RPMI 1640 medium supplemented with 100 IU/ml penicillin, 100 mg/ml streptomycin, 2 mM L-glutamine and 10% inactivated fetal calf serum (FCS) (Invitrogen, Paisley, UK). Tissue digestion was performed by adding 2 mg/ml collagenase D (Roche, Meylan, France), 1 mg/ml dispase (Invitrogen) and 0.1 mg/ml Dnase I (Roche). Digested PAR was crushed and filtered on 100 μm cell strainers. Red blood cells were lysed using erythrolysis buffer (10 mM NaHCO_3_, 155 mM NH_4_Cl, and 10 mM EDTA) for 10 min at 37°C. Cells were washed in PBS/EDTA and frozen in FCS + 10% dimethyl sulfoxide (DMSO, Sigma-Aldrich, St Louis, MO).

### *In vitro* infection

For *in vitro* experiments, lungs from conventionally bred Large White sows were obtained from a commercial slaughterhouse. All tested samples were negative for PRRSV. Cells were collected and processed as for *in vivo* experiments (section Virus Production and Titration). Cell preparations were then enriched in mononuclear phagocyte cells by 1.065 density iodixanol gradient (Optiprep®, Nycomed Pharma, Oslo, Norway) as previously described (25). These gradient-enriched mononuclear phagocyte cell preparations will be further referred as gradient-enriched MPC. Infections (FL13, LV, and Lena) were performed on fresh enriched MPC, 24 h, in complete RPMI at MOI 0.5 suitable for the detection of cell infection using the anti-N intracellular staining and FACS analysis (see below). In order to avoid MPC adherence, incubations at 37°C were performed in Corning-Costar 50 ml polypropylene centrifuge tubes, with the cap drilled for air renewal. Two complementary negative controls were performed, inactivated Lena virus incubated at 37°C and live LV, FL13, and Lena viruses incubated with cells at 4°C for the whole 24 h culture time.

For purified DC subtypes (cDC1, cDC2, moDC) and Macrophages (AM and AM-like) infection, cells were sorted as described in cell-sorting section (section Cell Sorting and Flow Cytometry Analysis). Then, Lena infections were performed in complete RPMI for 24 h at a multiplicity of infection (MOI) of 0.001, suitable for the detection of cell infection using the highly sensitive RT-qPCR method (section RNA Extraction and RT-qPCR on Sorted Cells).

### Cell sorting and flow cytometry analysis

Isolated BAL and PAR cells were stained in blocking solution, composed of PBS/EDTA supplemented with 5% Horse Serum and 5% Swine Serum. Antibodies were added to the blocking solution for 30 min on ice and then washed in PBS/EDTA with 2% FCS (for antibodies refer to Table 1). Staining were made in 4 steps, the uncoupled primary antibodies of different species/isotypes (Chicken anti-CadM1, mouse IgG1 anti-CD172a, mouse IgG2a anti-CD1, mouse IgG2b anti-MHC-II) followed by the secondary species/isotype specific fluorochrome-coupled antibodies (anti-chicken-Alexa647, anti-mouse IgG1-Alexa488, anti-mouse IgG2a-PE-Cy7, anti-mouse IgG2b APC-Cy7), then the fluorochrome-coupled or biotinylated primary antibodies anti-CD163-PE, biotinylated anti-CD11c) followed by the streptavidin-coupled Alexa700 fluorochrome. DAPI staining (Sigma-Aldrich) was performed to exclude dead cells. Compensations were set according to monocolor staining. When needed, fluorescence minus one (FMO) controls, using isotype control for the unstained channel, were used to ascertain the specificity of the staining (CD169, N, CD80/86, CD40, MHC-I, MHC-II expressions). MoFlo ASTRIOS sorter (Beckman-Coulter, Paris, France) was used to isolate specific cell subpopulations. FlowJo software (version 10.1.0, Tree Star, Ashland, OR) was used to analyze subpopulation prevalence. Population prevalence is expressed as percentage of MHC-II^high^/CD11c^pos^ cells. Cells were analyzed using an LSRFortessa cytometer and Diva software (Becton Dickinson, Franklin Lakes, New Jersey).

**Table 1:** Antibodies used for MPC cell sorting and infection analysis by flow cytometry.

**Antibody**	**Clone**	**Isotype**	**Specie**	**Working oncentrations**	**Supplier**
CadM1	3E1	IgY	Chicken	4 μg/ml	MBL
CD11c	3A8	IgG1	Mouse	2 μg/ml	Homemade
CD11c-biot	3A8	IgG1	Mouse	2 μg/ml	Homemade
CD163-PE	2A10/11	IgG1	Mouse	5 μg/ml	Biorad
CD169	1F1	IgG2a	Mouse	Pure (hybrifdoma supernatant)	Dr. J. Dominguez (INIA)
CD172a/Sirpα	74-22-15	IgG1	Mouse	4 μg/ml	WSU
	74-22-15a	IgG2b	Mouse	4 μg/ml	WSU
CD1	76-7-4	IgG2a	Mouse	8 μg/ml	WSU
CD1-FITC	76-7-4	IgG2a	Mouse	4 μg/ml	Southern Biotech
MHCII	MSA3	IgG2a	Mouse	4 μg/ml	WSU
MHCII	Th21A	IgG2b	Mouse	4 μg/ml	WSU
N	BIO276	IgG1	Mouse	Diluted 1/30	Bio-X Diagnostic

For intracellular virus detection, cells were stained using the same strategy as for cell-sorting. An additional intracellular staining step was performed using BD Cytofix/Cytoperm™ (Becton-Dickinson, Belgium), according to the manufacturer's instructions using an antibody against viral N protein (mouse IgG1, clone BIO 276, Bio-X Diagnostic) and a secondary anti-IgG1-PerCP-e710. In this case anti-MHC-II MSA3 (mouse IgG2a) and anti-CD172a 74-22-15a (mouse IgG2b) primary antibodies instead of Th21A and 74-22-15 respectively, as well as directly coupled CD1-FITC were used.

### RNA extraction and RT-qPCR on sorted cells

Total RNA from sorted cells was extracted using the Arcturus PicoPure RNA Isolation kit (ThermoFisher Scientific, St Aubin, France) according to the manufacturer's instructions. The RNase-Free DNase Set (Qiagen, France) was used to remove contaminating DNA. Reverse transcription was made using Multiscribe reverse transcriptase (ThermoFisher Scientific) according to manufacturer's instructions. Relative mRNA expression was evaluated by qPCR using the iTaq Universal SYBR Green Supermix (Biorad, Hercules, CA). Relative gene expression was assessed according to ΔCt method. *Ribosomal protein S24* (*RPS24*) and *Glyceraldehyde 3-phosphate dehydrogenase* (*GAPDH)* were used as reference genes as previously described in pig lung (21, 26). For viral transcripts, primers were designed on the Lena protein N nucleocapsid sequence. In all *in vivo* and *in vitro* infection, data were normalized on *RPS24* and *GAPDH* reference genes, previously tested for stable expression among MPC subsets and upon PRRSV infection (data not shown). Since normalization with *RPS24* or *GAPDH* gave similar results, only *RPS24* normalization is depicted in the Figures **4**–**6** and **Data Sheet 1**. Primers used are described in Table 2. Titration of viral particles using RT-qPCR detection, set a limit of detection < 1,000 particles/reaction (Ct < 35).

**Table 2:** Primers used for RT-qPCR.

**Gene**	**Sequences (5′-3′)**	**Gene ID**	**Size (bp)**	**Eff**	**R^2^**	**Slope**
*FcεRIα*	F: CAGGTGTCCTTGAATCCCCC	100152827	74	1.07	0.998	−3.06
	R: GGCATCTGTATTTGCCGCTG					
*FOXP3*	F: GGTGCAGTCTCTCTGGAACAA	444998	148	1.10	0.982	−3.10
	R: GGTGCCAGTGGCTACAATAC					
*GATA3*	F: GTCTAGCAAATCCAAAAAGTGCAA	733631	75	1	0.999	−3.32
	R: GGGTTGAACGAGCTGCTCTT					
*GAPDH*	F: CACCATCTTCCAGGAGCGAG	396823	51	1	0.998	−3.32
	R: CCAGCATCACCCCACTTGAT					
*IFNα*	F: TCTGCAAGGTTCCCAATGG	397686	69	0.93	0.999	−3.50
	R: GGCATTGCAGCTGAGTAGCA					
*IFNβ*	F: TGTGGAACTTGATGGGCAGA	445459	92	0.98	0.993	−3.22
	R: GAATGGTCATGTCTCCCCTGG					
*IFNγ*	F: TGGTAGCTCTGGGAAACTGAATG	396991	79	1.01	0.972	−3.30
	R: TGGCTTTGCGCTGGATCT					
*IL4*	F: GCCGGGCCTCGACTGT	397225	68	1.06	0.999	−3.18
	R: TCCGCTCAGGAGGCTCTTC					
*IL6*	F: CTGCTTCTGGTGATGGCTACTG	399500	69	0.94	0.983	−3.49
	R: GGCATCACCTTTGGCATCTT					
*Il8*	F: TCCTGCTTTCTGCAGCTCTCT	396880	71	0.72	0.991	−3.30
	R: GCACTGGCATCGAAGTTCTG					
*IL10*	F: GAGCCAACTGCAGCTTCCA	397106	65	1.01	0.991	−3.29
	R: TCAGGACAAATAGCCCACTAGCTT					
*IL12p35*	F: CGTGCCTCGGGCAATTATAA	397053	66	0.99	0.998	−3.34
	R: CAGGTGAGGTCGCTAGTTTGG					
*IL12p40*	F: GGAGCACCCCACATTCCTACT	397076	68	0.98	0.973	−3.36
	R: TTCTCTTTTGTTCTTGCCCTGAA					
*IL13*	F:CTGACCACCAGCATGCAGTACT	396721	59	0.93	0.979	−3.50
	R: CGCTGGCAGTCGGAGATGTT					
*IL17*	F: CCAGACGGCCCTCAGATTAC	449530	65	0.98	0.999	−3.72
	R: GGTCCTCGTTGCGTTGGA					
*IL21*	F: AAATAGTCATCTGCCTGATGGTCAT	403123	76	1	0.989	−3.30
	R: AGGCGATCTTGTCCTTGGAA					
*MerTK*	F: CCGAACTCTGTAATCGCTTCTTG	100519652	65	0.74	0.990	−3.16
	R: TGCACTTCCGCCGTGACTA					
*N* Lena	F: ATGGCCAGCCAGTCAATCAG	1494888	166	0.95	0.993	−3.46
	R: GGAACGTTCAGTTCCGGTGA					
*RORγt*	F: CCTGGCCCTGGGCATGT	100622477	138	1.09	0.908	−3.117
	R: TGTTCTAGCAGCGTCCGAAGT					
*RPS24*	F: AAGGAACGCAAGAACAGAATGAA	100155012	62	0.98	1	−3.37
ref. gene	R: TTTGCCAGCACCAACGTTG					
*T–bet*	F: TGCAGTCCCTCCATAAGTACCA	100518804	67	0.81	0.958	−3.32
	R: GCCTCTGGCTCACCATCATT					
*TGFβ*	F: GAAGCGCATCGAGGCCATTC	397078	162	0.85	0.999	−3.41
	R: GGCTCCGGTTCGACACTTTC					
*TNFα*	F: TGGTGGTGCCGACAGATG	397086	64	0.96	0.999	−3.42
	R: CAGCCTTGGCCCCTGAA					
*XCR1*	F: CGATGCCGTCTTCCACAAG	414375	61	1.01	0.989	−3.28
	R: GGAACCACTGGCGTTCTGA					

### Maturation markers surface expression

Expression of CD80/CD86, CD40, MHC-I, and MHC-II on DCs were studied by flow cytometry. Gradient-enriched MPC were cultured for 24 or 48 h with PRRSV (FL13, LV, and Lena) at MOI 0.5. For CD80/CD86 staining, the protein expressed by the human recombinant CD152 gene genetically fused with the Fc portion of the murine IgG2a sequence was used (Ancell, Bayport, MN, USA) (see Table 3).

**Table 3:** Antibodies used for maturation markers expressions and allogeneic lymphocytes proliferation assays.

**Antibody**	**Clone**	**Isotype**	**Specie**	**Dilution** **(μg/ml)**	**Supplier**
CD152-muIg (CTLA-4)		IgG2a	Mouse	10	Ancell
CD40	G28.5	IgG1	Mouse	10	Genetex
MHC-I	JM1E3	IgG1	Mouse	10	BioRad
CD3	8E6	IgG1	Mouse	10	WSU
CD4	74-12-4	IgG2b	Mouse	10	WSU

### Mixed lymphocytes reaction assay

Mixed Lymphocytes Reaction (MLR) assays were performed as previously described (27). Briefly, fresh peripheral blood mononuclear cells (PBMC) from Melanoma Libechov Mini pigs were isolated on Ficoll-Paque density gradient (Amersham Biosciences, Uppsala, Sweden). Fresh parenchymal gradient-enriched MPC were cultured at 1.10^6^ cells/ml for 24 h with PRRSV (FL13, LV and Lena) at MOI 0.5 in complete RPMI. Following tests at different MPC:PBMC ratio (from 1:2 to 1:12), the 1:6 ratio has been selected for fresh MPC:PBMC final co-culture, with final total cell concentration of 2 × 10^6^ per ml in complete RPMI. After 3 days of co-culture, total RNA was extracted and gene expression were analyzed by RT-qPCR. Four transcriptions factors (*T-bet, GATA3, ROR*γ*T*, and *FOXP3*) and cytokines (*IFN*γ, *IL13, IL17*, and *TGF*β) expression were chosen as indicators of T-helper polarization (respectively Th1, Th2, Th17, and Treg). *IL13* has been chosen instead of *IL4* because *IL13* has been reported as a better porcine Th2 marker than *IL4*(28). Data were normalized as described above in the RT-qPCR section. The same PBMC were CFSE stained and co-cultured with allogeneic infected gradient-enriched MPC in order to evaluate their proliferation rate. After 5 days of culture cells were stained using anti-CD3 and CD4 antibodies (Table 3) and their corresponding isotype-specific secondary antibodies and analyzed on LSR Fortessa flow cytometer.

### Cytokine bead assay

In order to measure production of cytokines by MPC in response to infection, gradient-enriched MPC were infected with PRRSV (FL13, LV and Lena) for 24 h at MOI 0.5 in complete RPMI and supernatants were collected. Cytokine production was measured by Cytokine Bead Assay (CBA) as previously described (21), using capture and detection antibodies combinations described in Table 4.

**Table 4:** Antibodies used for CBA.

**Cytokine**	**Capture antibodies**		**Detection antibodies (biotinylated)**	
	**Clone**	**Supplier**	**Clone**	**Supplier**
IL-1β	77724	R&DSystems	pAb (#BAF681)	R&DSystems
IL-2	A150D3F1	Invitrogen	A150D8H10	Invitrogen
IL-4	A155B16F2	Invitrogen	A155B15C6	Invitrogen
IL-6	pAb (#AF686)	R&DSystems	pAb (#BAF686)	R&DSystems
IL-8	105105	R&DSystems	pAb (#BAF535)	R&DSystems
IL-10	148801	R&DSystems	945A1A926C2	Invitrogen
IL-12	G9	KingfisherBiotech	116211	R&DSystems
IL-13	pAb (#PB0094S)	KingfisherBiotech	pAb (#PBB0096S)	KingfisherBiotech
IL-17	pAb (#KP0498S)	KingfisherBiotech	pAb (#KPB0499S)	KingfisherBiotech
TNFα	103104	R&DSystems	103302	R&DSystems
IFNα	F17	R&DSystems	K9	R&DSystems
IFNγ	A151D5B8	Invitrogen	A151D13C5	Invitrogen

### Statistical analysis

All data were analyzed using R software (version 3.4.0) upgraded with FactoMineR package (version 1.39) (29). Non-parametric approaches were chosen due to the few number of samples available. The Mann-Whitney's test was used to compare unpaired samples based on ranking. For paired samples, the Wilcoxon matched-pairs signed rank test was used. Statistical tests and *p*-values are indicated in the relevant figure legends. MPC cytokine profile after *in vivo* infection of pigs were analyzed using Principal Component Analysis method (PCA). PCA multivariate analysis is a dimension-reduction tool that permit to reduce a large set of variables to a smaller one which still contains most of the information. A first PCA analysis was performed on BAL and PAR subpopulations in order to validate subpopulation clusters. Regarding quality and quantity of RNA, only 3 out of 4 cell-sorting samples were included for cytokine expression analysis. A second PCA analysis was performed on cytokines expressions from the 3 DCs populations and the 2 tissues (PAR and BAL) separately.

## Results

### Dendritic cells are not infected *in vivo* and *in vitro* by LENA

**Figure 1:**
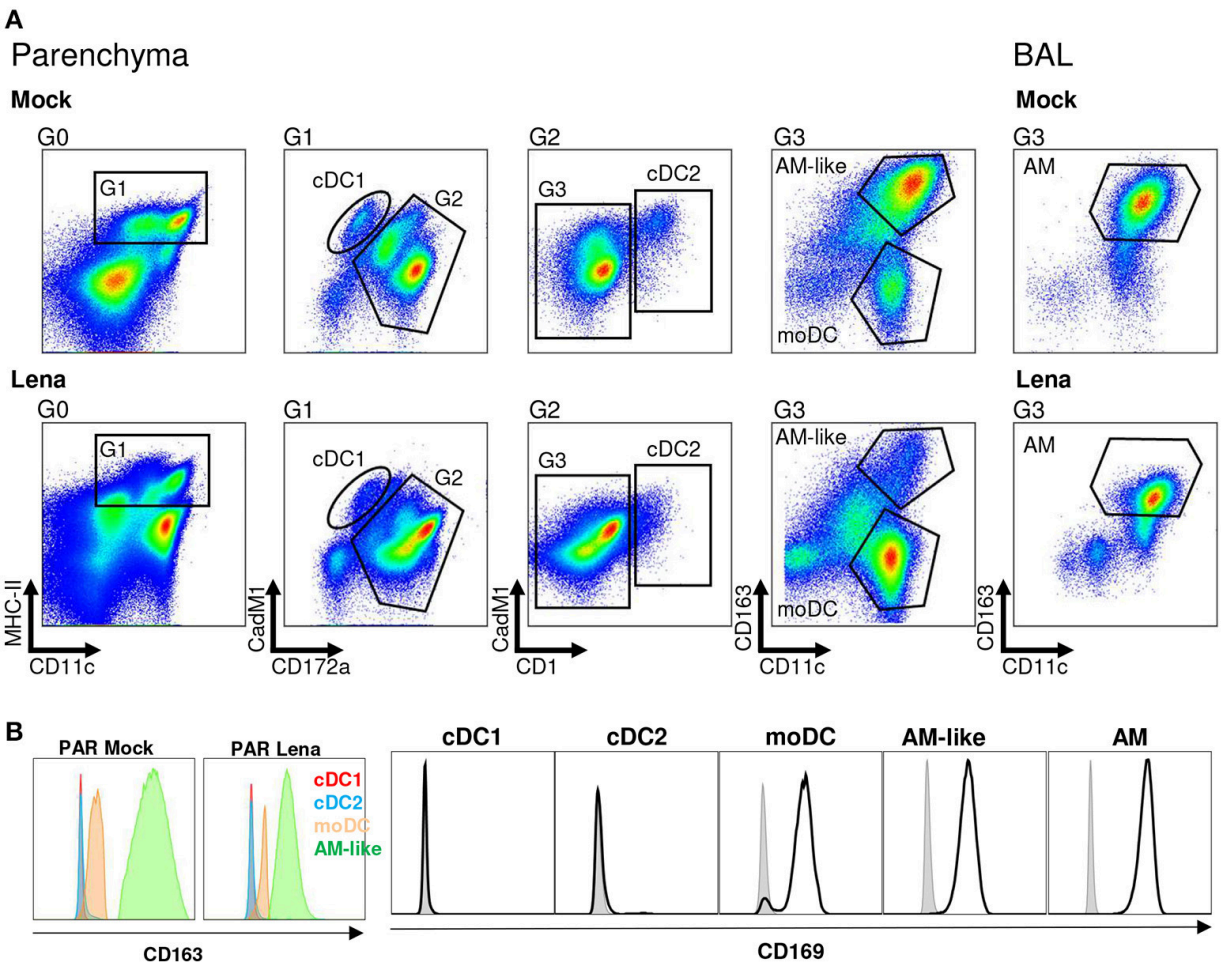
Mononuclear phagocytes (MPC) cell-sorting strategy. **(A)** Type 1 and Type 2 conventional dendritic cells (cDC1 and cDC2 respectively), monocyte-derived DCs (moDCs) and alveolar macrophages-like cells (AM-like) were isolated in infected or mock-infected lung parenchyma (PAR). MPC were defined by using MHC-II/CD11c/CD172a/CadM1/CD1 and CD163 markers. Identical process was performed on broncho-alveolar lavage (BAL) to collect alveolar macrophages (AM). Each graph is the offspring of the previous gate from left to right. Gates are numbered as G0 to G3. Data are representative of 4 independent cell-sorting experiments. **(B)** CD169 expression in MNP (Open black histogram) compared to isotype control (Closed gray histogram) and CD163 expression in MNP. Since CD163 is used in the gating of the different MNP populations, isotype control cannot be included.

**Figure 2:**
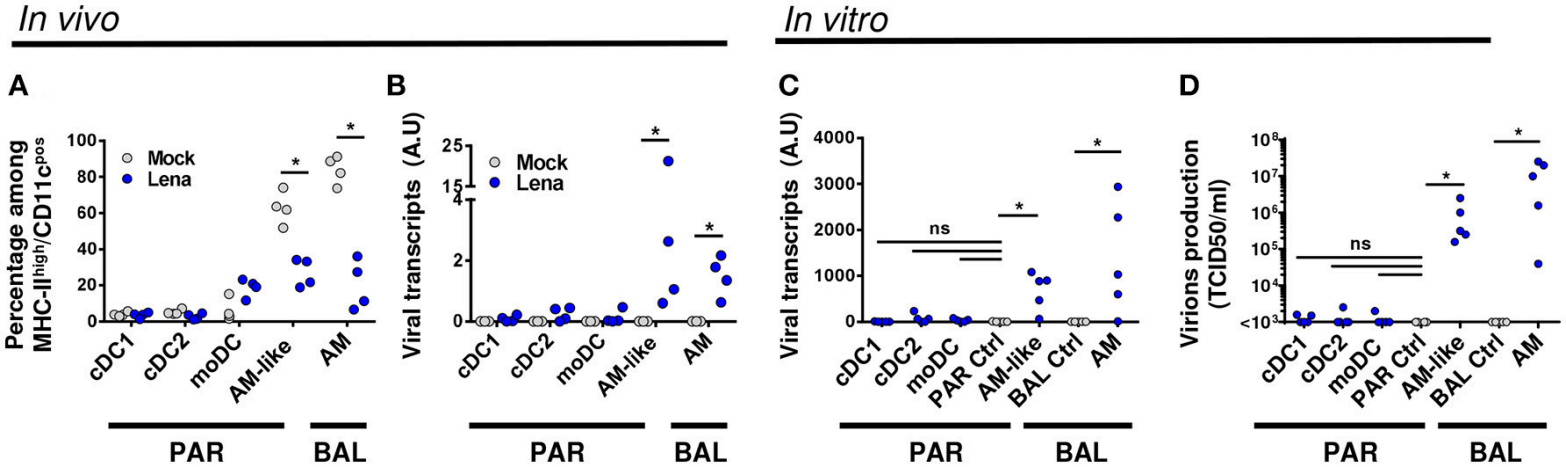
DCs are not infected by Lena *in vivo*. **(A)**. Percentage of MPC were monitored in mock (Gray circle, 4 animals) and infected (Blue circle, 4 animals) BAL and lung parenchyma (PAR) after immunostaining. Data are expressed as a percentage of MHC-II^high^/CD11c^pos^ cells (see Gate1 in Figure 1A). The Mann-Whitney's test was performed. ^*^p < 0.05. **(B)** Detection of viral transcripts on sorted MPC was assessed by RT-qPCR. Primers designed on viral Nucleocapsid protein (N) were used to detect viral transcripts. Arbitrary unit: A.U. represents 2^−ΔCt^. The Mann-Whitney's test was performed. ^*^p < 0.05. **(C,D)** MPC from conventional pigs were isolated by cell sorting and infected *in vitro* for 24 h with Lena at multiplicity of infection (MOI) 10^−3^. AM and AM-like cultivated with inactivated virus were defined as PAR and BAL controls (PAR Ctrl and BAL Ctrl) respectively. **(C)** Intracellular viral transcripts were detected by RT-qPCR. **(D)** Production of infectious virions were detected by titration of supernatants on fresh AM, according to TCID50/ml method. Data are representative of 5 independent experiments. Wilcoxon matched-pairs signed rank test was performed. ^*^p < 0.05.

Thanks to our previous work defining the phenotype and functions of the pig lung DC populations (cDC1, cDC2, and moDC) (21) we were able to refine our gating strategy in order to define each population by 4–5 different criterions, thus rendering our gating less sensitive to inflammation-induced markers modifications (Figure 1A). We used our newly described anti-CD11c antibody to gate myeloid cells (30). Conventional DC1 previously defined as MHC-II^high^/CD163^neg^/CD172a^neg/low^ will be defined here as MHC-II^high^/CD163^neg^/CD172a^neg/low^/CD11c^pos^/CadM1^pos^ (Figure 1A). Conventional DC2 previously defined as MHC-II^high^/CD163^neg^/CD172a^pos^ will be defined here as MHC-II^high^/CD163^neg^/CadM1^pos^/CD1^pos^ (Figure 1A) to comply with cDC2 definition in other tissues (27, 31). Conventional DC1 and cDC2 were negative for the two known PRRSV receptors CD163 and CD169 (Figure 1B), at steady state and upon infection. Monocyte derived-DC previously defined as MHC-II^high^/CD163^low^/CD172a^pos^ will be defined here as MHC-II^high^/CD163^low^/CD172a^pos^/CD11c^high^ (Figure 1A). CD163^high^/CD11c^high^ macrophages from BAL and parenchyma (PAR), called AM (21) and AM-like/PIM (21, 32) respectively were collected and used as positive control of infection in the following experiments. Monocyte-derived DC, AM-like/PIM and AM expressed CD169 and CD163 (Figure 1B), although moDC expressed lower level of CD163 as previously observed. AM (33) and PIM/AM-like (32) have been described to be the main PRRSV target cells, but they are unable to activate naïve T helper cells (21). This gating strategy was validated by cell-sorting of lung MPC upon mock and PRRSV infection and RT-qPCR of population specific genes (Supplementary Figure S1A). In both mock and infected animals, cDC1 exclusively presented a strong *XCR1* expression, whereas cDC2 presented a high *Fc*ε*RI*α expression, thus validating the cDC1 and cDC2 gating. We couldn't validate the moDCs sorting since no specific moDCs marker has been reported so far, neither in mouse (34–36) nor in human (37, 38) and swine (14, 21). As expected, *MerTK* was present in AM-like/PIM and AM. Surprisingly, in mock infected animals, steady state moDCs did not express *MertK*, whereas in our precedent experiments we observed variable *MertK* expression in moDC (21, 26). The discrepancy might originate from the use, here, of animals reared in highly controlled environment, whereas our previous studies were conducted using conventional animals. Indeed, upon Lena infection, we observed MertK upregulation in moDC (Supplementary Figure S1A), in concordance with a MertK upregulation in moDC in infectious conditions, although this hypothesis would need further validations. Since PRRSV is known to be cytolytic for infected cells, the evolution of each MPC population upon *in vivo* Lena infection was measured. Since the absolute number of MHC-II^high^/CD11c^pos^ MPC did not vary significantly among samples (PAR: 44.5.10^6^ ± 5.2.10^6^ cells in control animals vs. 40.8.10^6^ ± 4.6.10^6^ cells in infected animals; BAL: 44.1.10^6^ ± 9.2.10^6^ cells in control animals vs. 41.0.10^6^ ± 4.4.10^6^ cells in infected animals), the percentage of each MPC subtypes among MHC-II^high^/CD11c^pos^ cells was compared. Conventional DC1 and cDC2 proportions were not modified, whereas moDCs showed a trend to increase in parenchymal tissue (Figure 2A), and increased significantly in the alveolar lavages (Supplementary Figure S1B), in agreement with the pro-inflammatory infectious context. As previously observed (32), AM-like and AM proportions were strongly decreased (Figure 1A). The same cell populations were then sorted and viral RNAs were detected using RT-qPCR. The clear decrease of AM and AM-like proportions was concomitant with the detection of viral RNA. No significant viral RNA was observed in the 3 parenchymal DCs populations (Figure 2B) as well as in their alveolar counterparts (Supplementary Figure S1C).

To comfort this *in vivo* results, we cell-sorted the MPC of uninfected animals, and infected the cell populations *in vitro* at low MOI. AM and AM-like cultured with inactivated Lena were used as negative control for BAL and PAR respectively. After 2 days of culture, viral transcripts in the cell pellet as well as infectious virions in the culture supernatant were titrated by RT-qPCR and TCID50 methods respectively. Whereas AM-like and AM gave clear positive signals, no significant differences were observed between DCs and the negative controls with both detection methods (Figures 2C,D). This set of data clearly demonstrated, both *in vivo* and *in vitro*, that none of the 3 subsets of DCs were infected by Lena.

### DCs are not infected *in vitro* by FL13 and LV

**Figure 3:**
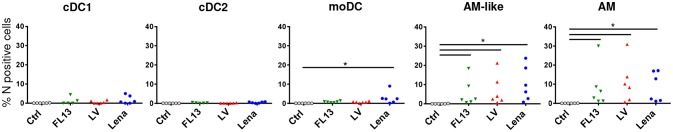
DCs are not infected by Lelystad and Flanders13 *in vitro*. MPC enriched parenchymal cells were infected for 16 h at MOI 0.5 with Flanders13 (FL13, Green triangle), Lelystad (LV, Red triangle), and Lena (Blue circle) strains. Mock-infected MPC were used as control (Ctrl). The cells were stained and gated as in Figure 1, except the additional step of an intracellular viral N protein staining. Data are representative of 6 independent experiments. Wilcoxon matched-pairs signed rank test was performed. ^*^p < 0.05.

Lena tropism was compared with that of FL13 and LV. An original *in vitro* model of infection using gradient enriched MPC was established. This method allows to keep the lung MPC complexity in an easy to handle cell suspension culture. After 24 h (an incubation time at which the cytopathic effect of PRRSV infection is not yet observed), viral infection was detected using an intracellular staining of viral N protein associated with the previously used MPC staining and gating (Figure 1). This model was validated by performing Lena infection and asserting the correlation between the levels of viral RNA by RT-qPCR (Figure 2) and N intracellular staining (data not shown). Controls with inactivated virus and 4°C incubated virus were performed (Supplementary Figure S2). For all strains, AM and AM-like presented N protein expression (Figure 3), validating the replication previously observed by RT-qPCR. In line with the previous *in vivo* and *in vitro* data, the viral protein was not detected in cDCs neither in PAR (Figure 3) nor in BAL (data not shown) for the 3 strains, in agreement with their lack of CD163 expression. In contrast with our *in vivo* and *in vitro*, cell sorted infection data (Figures 2A,B); using MPC mixed *in vitro* culture, a small but significant proportion (under 5%) of moDCs were N positive upon Lena, but not LV and FL13 infections (Figure 3 and Supplementary Figure S2).

### Lena but not FL13 and LV triggers a Th1 response *in vitro*

The resistance of DCs to PRRSV infection did not rule out a functional impairment of DCs by PRRSV, either through an early abortive infection or by an indirect bystander action. In order to test DCs functionalities upon PRRSV-1.1 and 1.3 infections, we used our *in vitro* infectious system to probe the complex action of the 3 strains on the whole lung MPC.

**Figure 4:**
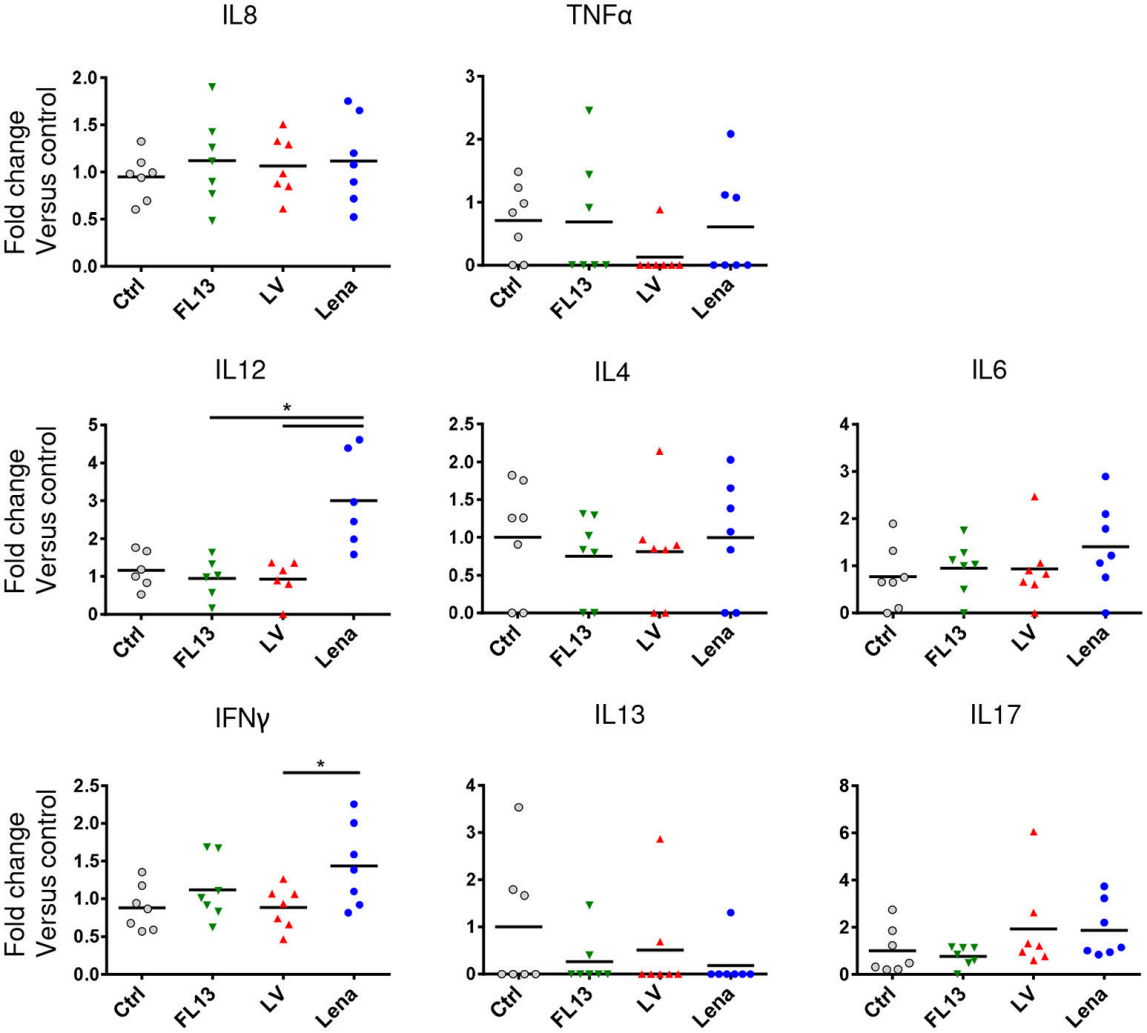
MPC exposure to Lena virus induces IL12A and IFNg secretions. MPC enriched PAR were cultured for 24 h at MOI 0.5 with FL13 (Green triangle), LV (Red triangle), and Lena (Blue circle) in complete RPMI. Supernatants were collected and analyzed by Cytokine Bead Assay (CBA). Data were normalized as fold change relative to control. The control samples mean has been calculated for each cytokine, then, each sample value, including the control sample values used for the reference mean calculation, is individually expressed as a percentage of this mean. Wilcoxon test was performed. ^*^p < 0.05.

Production of cytokines by parenchymal MPC after infection was evaluated by CBA. IL12 and at a lesser extend IFNγ releases were significantly increased upon Lena but not LV and FL13 infections (Figure 4). No significant differences between viruses were observed for IL8, TNFα, IL4, IL6, IL13, and IL17. Maturation of DCs were tested *in vitro* by monitoring CD80/86, CD40, MHC-I, and MHC-II expressions by FACS analysis. No difference between strains appeared in these experiments (Supplementary Figure S3A).

**Figure 5:**
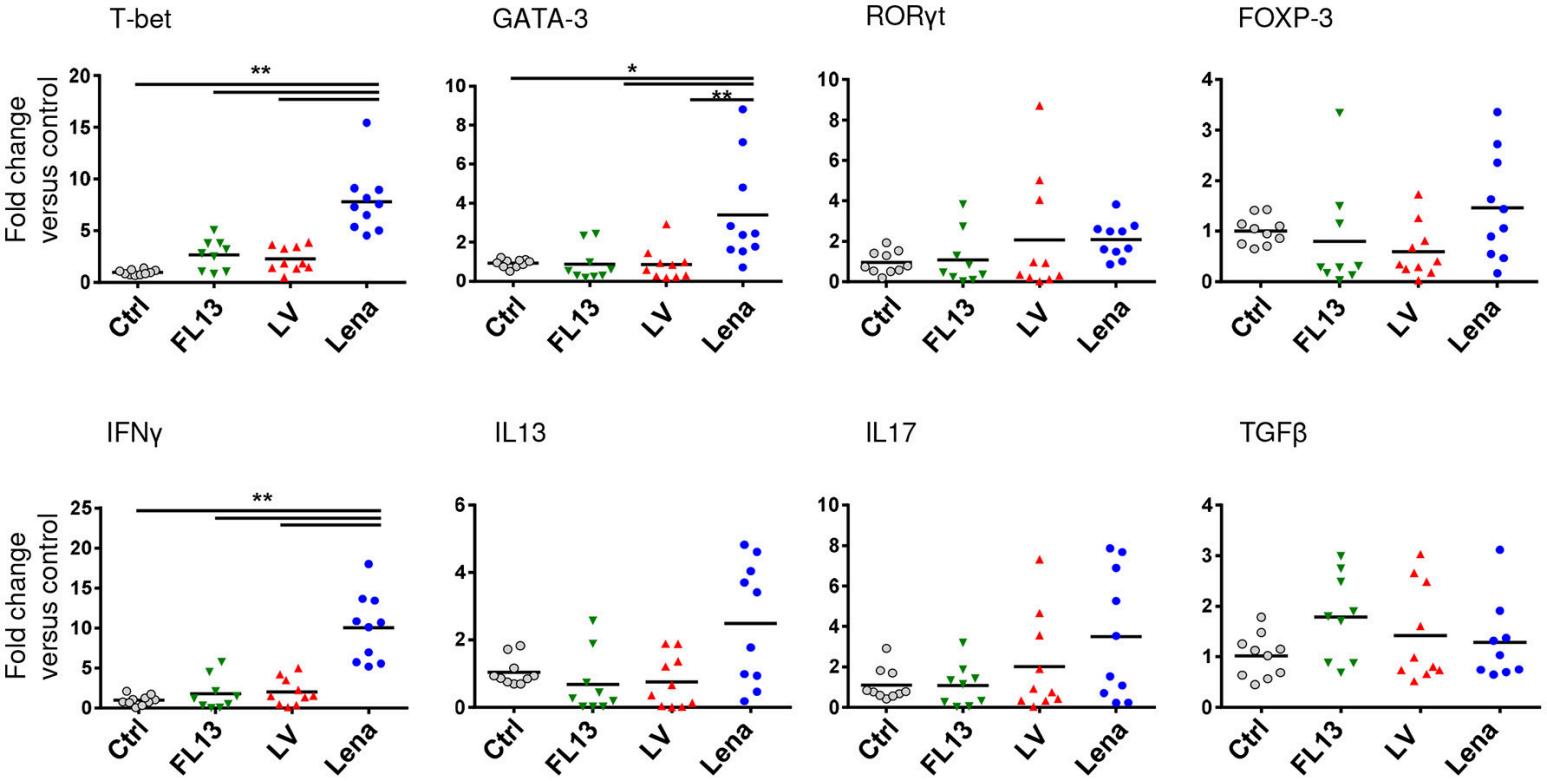
Lena infected MPC trigger a stronger Th1 response than LV and FL13. MPC-enriched PAR were cultured for 24 h with FL13 (Green triangle), LV (Red triangle), or Lena (Blue circle) at MOI 0.5 and then co-cultured with allogeneic PBMC at ratio 1:6. After 3 days of co-culture, RNA were extracted and gene expressions were analyzed by RT-qPCR. Four transcriptions factors (*T-bet, GATA3, ROR*γ*T*, and *FOXP3*) and cytokines (*IFN*γ, *IL13, IL17*, and *TGF*β) expressions were chosen as indicator of respectively Th1, Th2, Th17, and Treg polarization. Data were normalized to the reference gene *RPS24* expression and expressed as fold change relative to control. The control samples mean has been calculated for each cytokine, then, each control sample is expressed as a percentage of this mean. Wilcoxon test was performed. ^*^p < 0.05; ^**^ < 0.005.

In order to analyze the ability of the pooled infected MPC to induce a T cell response, MLR assays were performed. MPC infected with any of the 3 strains induced a similar proliferation of CD4 T cells (Supplementary Figure S3B) in agreement with the absence of maturation markers expression differences. Using the same settings, we investigated more subtle functional differences by looking at the induction of T helper (Th) differentiation bias by measuring transcription factors and cytokines mRNA expressions after 3 days of allogeneic culture. Contrary to LV and FL13 infected MPC, Lena infected MPC showed a clear Th1 profile characterized by a higher expression of *T-bet* and *IFN*γ (Figure 5), in agreement with the detection of IL12 and IFNγ cytokines. Lena infection also triggered a significant *GATA-3* expression, paralleled with a non-significant *IL13* upregulation. Interestingly, in our settings no Treg nor Th17 induction was observed with any of the 3 strains.

### Lena infection *in vivo* activates the Th1-inducer cDC1

Since DCs are the only activator of naïve T cells, and given the clear Th1 profile detected with Lena infection *in vitro*, we investigated DCs subpopulations' activation *in vivo*. We assessed the cytokines mRNA expression levels of sorted DCs from 10 days mock and Lena infected animals. The expressions data were analyzed in a first PCA (Supplementary Figure S4). MPC subpopulations clearly segregated on the two first axis regardless of tissue (PAR vs. BAL) and infection status, thus clearly validating our sorting strategy.

**Figure 6:**
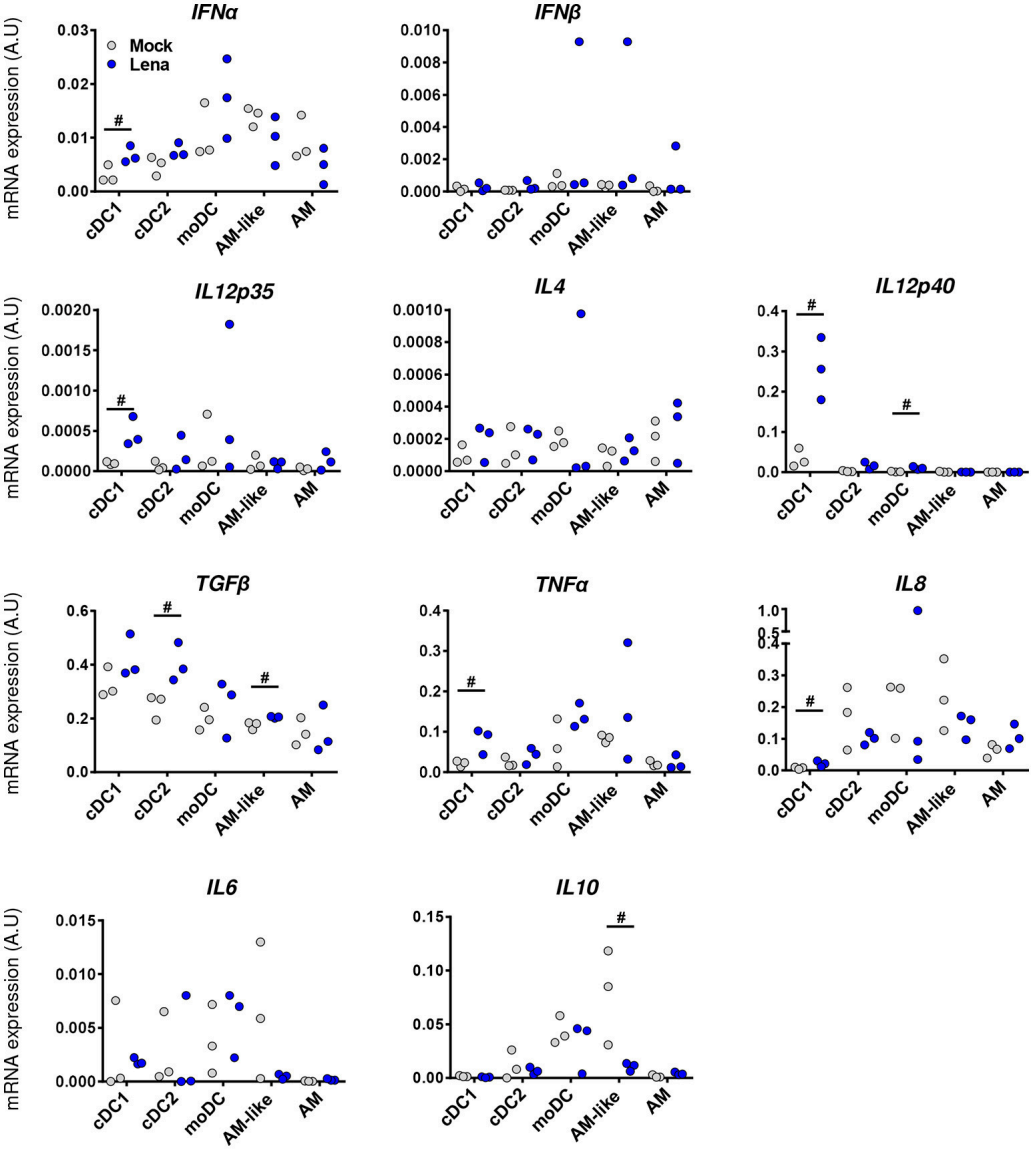
Lena infection triggers differential cytokines responses according to DCs subpopulations. Cytokines mRNA expressions in MPC from mock (Gray Dots) or *in vivo* infected (Blue Dots) PAR and BAL were studied by RT-qPCR after cell-sorting. Data were normalized to the reference gene *RPS24* expression. Data are representative of 3 independent experiments. Ranking tests were performed. Symbol “#” highlights when the infected animals were all upper or lower than the controls.

**Figure 7:**
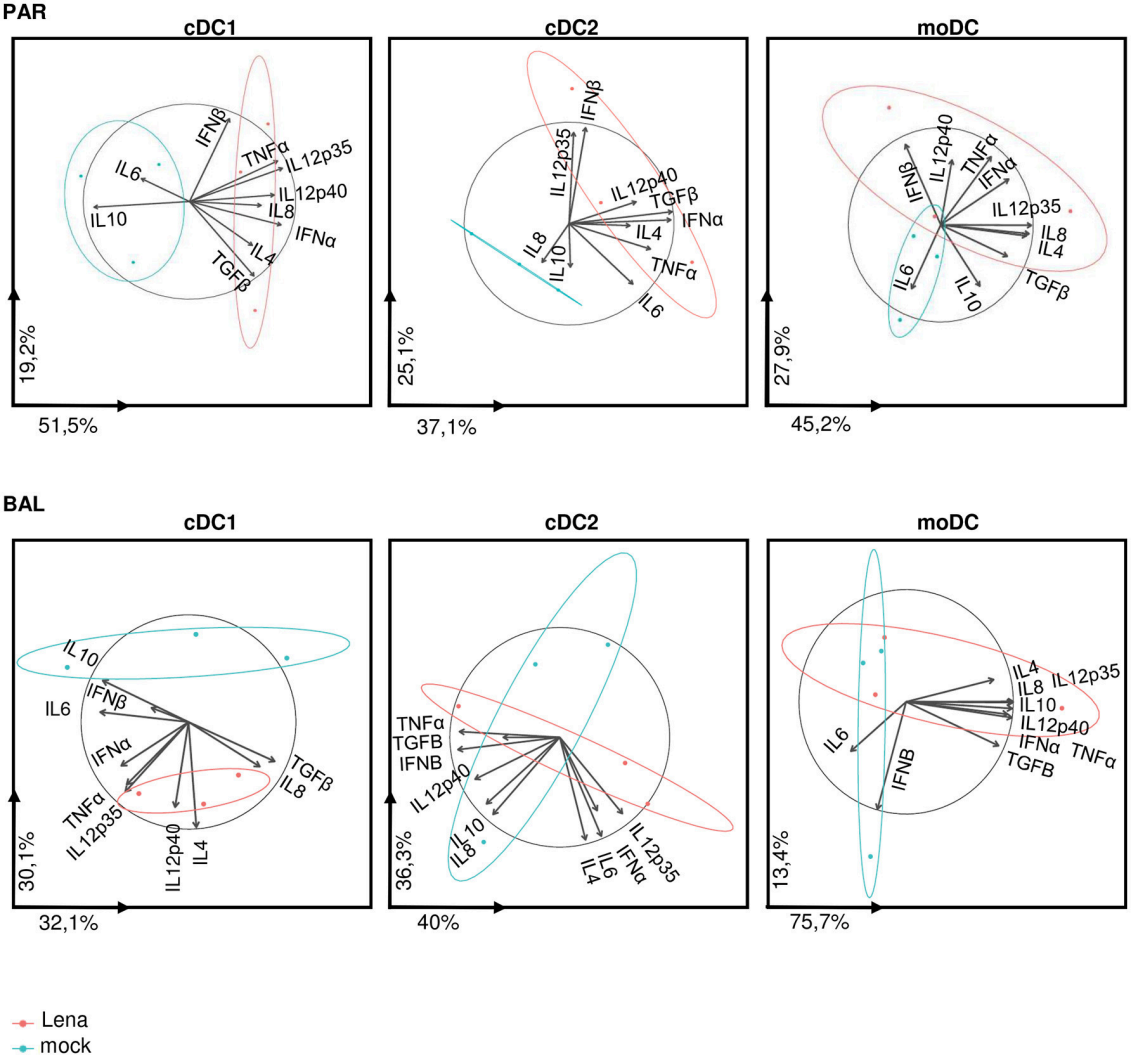
cDC1 are activated upon *in vivo* Lena infection. Principal Component Analysis (PCA) of DCs cytokines profile from PAR and BAL. The parameter values are cytokine mRNA expressions of sorted DCs from *in vivo* infected animals (Figure 6). Each pig is represented as a dot of specific color according to its group Mock (blue) or Lena (red). The percentages indicated on the axis represents the variance between individuals explain by this axis. The PCA was done with 10 factors (each of one represent a cytokine). PCA graphics has been produced using FactoMineR R package (version 1.39) (29). The ellipses depict the spread of the PCA data for mock or Lena infected conditions (blue and red respectively). Non-overlapping ellipses attest a significant difference in the global cytokine profile between mock and infected samples.

Analysis of individual cytokines expression indicated trends of upregulation in cells from Lena infected animals for *IFN*α, *IL12p35, IL12p40, IL8*, and *TNF*α in cDC1, upregulation of *TGF*β in cDC2, and upregulation of *IL12p40* in moDCs. In AM-like *TGF*β was upregulated and *IL10* was down-regulated (Figure 6). These data were then analyzed globally in a second PCA analysis which showed a clear change in cDC1 cytokines profile under infectious conditions in PAR and BAL samples (Figure 7). For cDC2 and moDCs no clear differences were observed. It should be noticed that the same analysis in AM and AM-like gave no clear separation between cells from mock or infected animals (data not shown).

## Discussion

Here, *in vitro* and *in vivo* experiments were developed to study the interactions of lung DCs with different PRRSV-1. Our results demonstrate that the two types of cDCs (cDC1 and cDC2) are not infected by PRRSV-1. Using the MPC mix culture model, moDCs appeared susceptible, although weakly, to Lena, but not to PRRSV-1.1 infections whereas *in vivo* infected and freshly purified moDCs were not. One possible explanation is that monocytes present in lung MPC became sensitive to Lena infection upon culture as previously described for PRRSV2 (39) and then differentiated in moDCs in the course of *in vitro* culture in inflammatory conditions. This would explain contradictory results reported according to Lena infections experiments using MPC *ex vivo* or *in vitro* (14, 15, 40) as they might greatly depend upon the *in vitro* culture conditions. Although we were unable to identify and isolate lung plasmacytoid DCs (pDCs), a previous report on blood pDCs demonstrated that pDCs were also resistant to PRRSV infections (41). Thus, according to our *in vivo* and *in vitro* data, we can confidently state that none of the known DCs subtypes of the porcine lung can be consistently infected by PRRSV-1.

The resistance to PRRSV infection of *in vivo* differentiated cDCs appears in line with the absence of expression in these cells of the two main PRRSV receptors CD169 and CD163 (Figure 1) (21). To note, Siglec-10 has been recently described as a possible alternative to CD169 (Siglec-1) receptor (42). However Siglec-10 expression seems restricted to B cells (43), and would not compensate for the absence of CD163 expression on cDCs, CD163 being so far the only receptor whose deletion totally abrogates *in vivo* PRRSV replication (44, 45). Conversely, *in vivo* differentiated moDCs expressed CD169 and low levels of CD163 [Figure 1; (21)] that might be upregulated upon *in vitro* culture, which would explain the infection of moDCs depending on culture conditions and PRRSV type.

In Lena infected animals, the absence of DCs infection did not preclude the cDC1 activation and the development of an antiviral Th1 immune response. This is in agreement with the role of cDC1 in the antiviral Th1/T CD8 inducing response, as previously observed in porcine (21) and murine (CD103^pos^ DCs) (46, 47) respiratory tracts. Lung mouse cDC1 (CD103^+^) have been shown to be resistant to Influenza virus infection while cross-presenting efficiently viral antigens to T cells (48). Moreover it has been recently demonstrated that human cDC1 were resistant to infection by endocytic enveloped viruses such as HIV and Influenza but relied on viral antigens produced by bystander cells that they can cross-present to trigger T cell responses (49). In the case of PRRSV, those antigens might come from apoptotic infected macrophages. Indeed cDC1 from murine lung (CD103^pos^ DCs) (50) and from pig skin (CD172a^neg/low^ dermal DCs)(25) preferentially phagocytosed apoptotic bodies. Activation of porcine cDC1 can be triggered through TLR8 (31) which is activated by single-strand RNA viruses (51) or through the accumulation of late apoptotic/secondary necrotic, pro-inflammatory AM that might accumulate in the lung [for review see (52)]. This bystander activation of cDC1 can be compared to the previously described *in vitro* activation of pDCs (53) by PRRSV-infected macrophages. We can thus speculate that Lena-infected apoptotic AM are taken over by cDC1 that get activated by virus-laden apoptotic bodies. Conventional cDC1 then activate the Th1/TCD8 immune responses. We have to stress here that our data used allogeneic induced responses, but did not formally demonstrate PRRSV-specific antigen presentations. However, interestingly, the Th1 immune response induced by PRRSV-1.3 has been correlated with the clinical score (5) suggesting that the antigen-specific anti-PRRSV Th1 response would be involved in the higher pathogenicity of PRRSV-1.3 strains. Whereas this Lena-triggered cDC1 activation appears finally quite classical, we would like to stress that the converse lack of cDC1/Th1 activation with PRRSV-1.1 viruses might indeed deserve more attention.

## Author contributions

ElB, EC, EdB, PM, and NB processed the samples and performed the *in vitro* and *ex vivo* experiments. FB performed the CBA analysis. PR, J-JL, and OB performed the *in vivo* experiments. ElB, MT, and LJ performed the statistical analysis. ElB and MB performed the cell-sorting. EG and IS-C provided thorough discussions and critical manuscript reading. OB supervised the *in vivo* experiments. LJ supervised the statistical analysis. EG, IS-C, OB, and NB provided financial supports. NB supervised the working program. ElB and NB wrote the manuscript.

### Conflict of interest statement

The authors declare that the research was conducted in the absence of any commercial or financial relationships that could be construed as a potential conflict of interest.

## Abbreviations

AM: Alveolar Macrophages
AM-like: Alveolar Macrophages like
BAL: Broncho-Alveolar Lavage
cDC1: conventional Dendritic Cells type 1
cDC2: conventional Dendritic Cells type 2
CBA: Cytokine Bead Assay
DCs: Dendritic Cells
FL13: Flanders13 virus
LV: Lelystad virus
moDC: Monocyte-derived Dendritic Cells
MPC: Mononuclear Phagocyte Cells
MP: Multiplicity Of Infection
PAR: Parenchyma
pDCs: plasmacytoid Dendritic Cells
PRRSV: Porcine Reproductive and Respiratory Syndrome Virus
PCA: Principal Component Analysis.

## References

[1] Kuhn JH Lauck M Bailey AL Shchetinin AM Vishnevskaya TV Bao Y. Reorganization and expansion of the nidoviral family *Arteriviridae*. Arch Virol. (2016) 161:755–68. 10.1007/s00705-015-2672-z 26608064 PMC5573231

[2] Stadejek T Stankevicius A Murtaugh MP Oleksiewicz MB. Molecular evolution of PRRSV in Europe: current state of play. Vet Microbiol. (2013) 165:21–8. 10.1016/j.vetmic.2013.02.029 23528651

[3] Forsberg R Storgaard T Nielsen HS Oleksiewicz MB Cordioli P Sala G. The genetic diversity of European type PRRSV is similar to that of the North American type but is geographically skewed within Europe. Virology (2002) 299:38–47. 10.1006/viro.2002.1450 12167339

[4] Garcia-Nicolas O Baumann A Vielle NJ Gomez-Laguna J Quereda JJ Pallares FJ. Virulence and genotype-associated infectivity of interferon-treated macrophages by porcine reproductive and respiratory syndrome viruses. Virus Res. (2014) 179:204–11. 10.1016/j.virusres.2013.08.009 24220223

[5] Morgan SB Graham SP Salguero FJ Sanchez Cordon PJ Mokhtar H Rebel JM. Increased pathogenicity of European porcine reproductive and respiratory syndrome virus is associated with enhanced adaptive responses and viral clearance. Vet Microbiol. (2013) 163:13–22. 10.1016/j.vetmic.2012.11.024 23313323

[6] Weesendorp E Morgan S Stockhofe-Zurwieden N Popma-De Graaf DJ Graham SP Rebel J. M. Comparative analysis of immune responses following experimental infection of pigs with European porcine reproductive and respiratory syndrome virus strains of differing virulence. Vet Microbiol. (2013) 163:1–12. 10.1016/j.vetmic.2012.09.013 23036445 PMC7117209

[7] Renson P Rose N Le Dimna M Mahe S Keranflec'h A Paboeuf F. Dynamic changes in bronchoalveolar macrophages and cytokines during infection of pigs with a highly or low pathogenic genotype 1 PRRSV strain. Vet Res. (2017) 48:15. 10.1186/s13567-017-0420-y 28241868 PMC5327547

[8] Butler JE Lager KM Golde W Faaberg KS Sinkora M Loving C. Porcine reproductive and respiratory syndrome (PRRS): an immune dysregulatory pandemic. Immunol Res. (2014) 59:81–108. 10.1007/s12026-014-8549-5 24981123 PMC7091131

[9] Rahe MC Murtaugh M. P. Mechanisms of adaptive immunity to porcine reproductive and respiratory syndrome virus. Viruses (2017) 9:E148. 10.3390/v9060148 28608816 PMC5490824

[10] Mardassi H Wilson L Mounir S Dea S. Detection of porcine reproductive and respiratory syndrome virus and efficient differentiation between Canadian and European strains by reverse transcription and PCR amplification. J Clin Microbiol. (1994) 32:2197–203. 7814546 10.1128/jcm.32.9.2197-2203.1994 PMC263966

[11] Van Gorp H Van Breedam W Delputte PL Nauwynck HJ. Sialoadhesin and CD163 join forces during entry of the porcine reproductive and respiratory syndrome virus. J Gen Virol. (2008) 89:2943–53. 10.1099/vir.0.2008/005009-0 19008379

[12] Silva-Campa E Cordoba L Fraile L Flores-Mendoza L Montoya M Hernandez J European genotype of porcine reproductive and respiratory syndrome (PRRSV) infects monocyte-derived dendritic cells but does not induce Treg cells. Virology (2010) 396:264–71. 10.1016/j.virol.2009.10.024 19913865

[13] Weesendorp E Stockhofe-Zurwieden N Popma-De Graaf DJ Fijten H Rebel JM. Phenotypic modulation and cytokine profiles of antigen presenting cells by European subtype 1 and 3 porcine reproductive and respiratory syndrome virus strains *in vitro* and *in vivo*. Vet Microbiol. (2013) 167:638–50. 10.1016/j.vetmic.2013.09.021 24120935

[14] Singleton H Graham SP Bodman-Smith KB Frossard JP Steinbach F. Establishing porcine monocyte-derived macrophage and dendritic cell systems for studying the interaction with PRRSV-1. Front Microbiol. (2016) 7:832. 10.3389/fmicb.2016.00832 27313573 PMC4889594

[15] Frydas IS Verbeeck M Cao J Nauwynck HJ. Replication characteristics of porcine reproductive and respiratory syndrome virus (PRRSV) European subtype 1 (Lelystad) and subtype 3 (Lena) strains in nasal mucosa and cells of the monocytic lineage: indications for the use of new receptors of PRRSV (Lena). Vet Res. (2013) 44:73. 10.1186/1297-9716-44-73 24007551 PMC3849772

[16] van Furth R Cohn ZA Hirsch JG Humphrey JH Spector WG Langevoort HL. The mononuclear phagocyte system: a new classification of macrophages, monocytes, and their precursor cells. Bull World Health Organ. (1972) 46:845–52. 4538544 PMC2480884

[17] Proll MJ Neuhoff C Schellander K Uddin MJ Cinar MU Sahadevan S. Transcriptome profile of lung dendritic cells after *in vitro* porcine reproductive and respiratory syndrome virus (PRRSV) infection. PLoS ONE (2017) 12:e0187735. 10.1371/journal.pone.0187735 29140992 PMC5687707

[18] Loving CL Brockmeier SL Sacco RE. Differential type I interferon activation and susceptibility of dendritic cell populations to porcine arterivirus. Immunology (2007) 120:217–29. 10.1111/j.1365-2567.2006.02493.x 17116172 PMC2265861

[19] Guilliams M Ginhoux F Jakubzick C Naik SH Onai N Schraml BU. Dendritic cells, monocytes and macrophages: a unified nomenclature based on ontogeny. Nat Rev Immunol. (2014) 14:571–8. 10.1038/nri3712 25033907 PMC4638219

[20] Guilliams M van de Laar L. A Hitchhiker's guide to myeloid cell subsets: practical implementation of a novel mononuclear phagocyte classification system. Front Immunol. (2015) 6:406. 10.3389/fimmu.2015.00406 26322042 PMC4531301

[21] Maisonnasse P Bouguyon E Piton G Ezquerra A Urien C Deloizy C. The respiratory DC/macrophage network at steady-state and upon influenza infection in the swine biomedical model. Mucosal Immunol. (2016) 9:835–49. 10.1038/mi.2015.105 26530136

[22] Karniychuk UU Geldhof M Vanhee M Van Doorsselaere J Saveleva TA Nauwynck HJ. Pathogenesis and antigenic characterization of a new East European subtype 3 porcine reproductive and respiratory syndrome virus isolate. BMC Vet Res. (2010) 6:30. 10.1186/1746-6148-6-30 20525333 PMC2898778

[23] Wensvoort G Terpstra C Pol JM ter Laak EA Bloemraad M de Kluyver EP. Mystery swine disease in The Netherlands: the isolation of Lelystad virus. Vet Q. (1991) 13:121–30. 10.1080/01652176.1991.9694296 1835211

[24] Renson P Fablet C Le Dimna M Mahe S Touzain F Blanchard Y. Preparation for emergence of an Eastern European porcine reproductive and respiratory syndrome virus (PRRSV) strain in Western Europe: immunization with modified live virus vaccines or a field strain confers partial protection. Vet Microbiol. (2017) 204:133–40. 10.1016/j.vetmic.2017.04.021 28532792

[25] Marquet F Bonneau M Pascale F Urien C Kang C Schwartz-Cornil I. Characterization of dendritic cells subpopulations in skin and afferent lymph in the swine model. PLoS ONE (2011) 6:e16320. 10.1371/journal.pone.0016320 21298011 PMC3029332

[26] Maisonnasse P Bordet E Bouguyon E Bertho N. Broncho alveolar dendritic cells and macrophages are highly similar to their interstitial counterparts. PLoS ONE (2016) 11:e0167315. 10.1371/journal.pone.0167315 27992536 PMC5167224

[27] Marquet F Vu Manh TP Maisonnasse P Elhmouzi-Younes J Urien C Bouguyon E. Pig skin includes dendritic cell subsets transcriptomically related to human CD1a and CD14 dendritic cells presenting different migrating behaviors and T cell activation capacities. J Immunol. (2014) 193:5883–93. 10.4049/jimmunol.1303150 25385823

[28] Murtaugh MP Johnson CR Xiao Z Scamurra RW Zhou Y. Species specialization in cytokine biology: is interleukin-4 central to the T(H)1-T(H)2 paradigm in swine? Dev Comp Immunol. (2009) 33:344–52. 10.1016/j.dci.2008.06.014 18761033

[29] Lê S Josse J Husson F FactoMineR: an R package for multivariate analysis. J.Stat Softw. (2008) 25:1–18. 10.18637/jss.v025.i01

[30] Deloizy C Bouguyon E Fossum E Sebo P Osicka R Bole A. Expanding the tools for identifying mononuclear phagocyte subsets in swine: reagents to porcine CD11c and XCR1. Dev Comp Immunol. (2016) 65:31–40. 10.1016/j.dci.2016.06.015 27345169

[31] Auray G Keller I Python S Gerber M Bruggmann R Ruggli N. Characterization and transcriptomic analysis of porcine blood conventional and plasmacytoid dendritic cells reveals striking species-specific differences. J Immunol. (2016) 197:4791–806. 10.4049/jimmunol.1600672 27837108

[32] Bordet E Maisonnasse P Renson P Bouguyon E Crisci E Tiret M. Porcine alveolar macrophage-like cells are pro-inflammatory pulmonary intravascular macrophages that produce large titers of porcine reproductive and respiratory syndrome virus. Sci Rep. (2018) 8:10172. 10.1038/s41598-018-28234-y 29977043 PMC6033929

[33] Duan X Nauwynck HJ Pensaert MB. Virus quantification and identification of cellular targets in the lungs and lymphoid tissues of pigs at different time intervals after inoculation with porcine reproductive and respiratory syndrome virus (PRRSV). Vet Microbiol. (1997) 56:9–19. 10.1016/S0378-1135(96)01347-8 9228678

[34] Hammad H Plantinga M Deswarte K Pouliot P Willart MA Kool M. Inflammatory dendritic cells–not basophils–are necessary and sufficient for induction of Th2 immunity to inhaled house dust mite allergen. J Exp Med. (2010) 207:2097–111. 10.1084/jem.20101563 20819925 PMC2947072

[35] Langlet C Tamoutounour S Henri S Luche H Ardouin L Gregoire C. CD64 expression distinguishes monocyte-derived and conventional dendritic cells and reveals their distinct role during intramuscular immunization. J Immunol. (2012) 188:1751–60. 10.4049/jimmunol.1102744 22262658

[36] Plantinga M Guilliams M Vanheerswynghels M Deswarte K Branco-Madeira F Toussaint W. Conventional and monocyte-derived CD11b(+) dendritic cells initiate and maintain T helper 2 cell-mediated immunity to house dust mite allergen. Immunity (2013) 38:322–35. 10.1016/j.immuni.2012.10.016 23352232

[37] Cheong C Matos I Choi JH Dandamudi DB Shrestha E Longhi MP. Microbial stimulation fully differentiates monocytes to DC-SIGN/CD209(+) dendritic cells for immune T cell areas. Cell (2010) 143:416–29. 10.1016/j.cell.2010.09.039 21029863 PMC3150728

[38] Segura E Touzot M Bohineust A Cappuccio A Chiocchia G Hosmalin A. Human inflammatory dendritic cells induce Th17 cell differentiation. Immunity (2013) 38:336–48. 10.1016/j.immuni.2012.10.018 23352235

[39] Wang L Zhang H Suo X Zheng S Feng WH Increase of CD163 but not sialoadhesin on cultured peripheral blood monocytes is coordinated with enhanced susceptibility to porcine reproductive and respiratory syndrome virus infection. Vet Immunol Immunopathol. (2011) 141:209–20. 10.1016/j.vetimm.2011.03.001 21440313

[40] Singleton H Graham SP Frossard JP Bodman-Smith KB Steinbach F. Infection of monocytes with European porcine reproductive and respiratory syndrome virus (PRRSV-1) strain Lena is significantly enhanced by dexamethasone and IL-10. Virology (2018) 517:199–207. 10.1016/j.virol.2018.02.017 29502802

[41] Baumann Mateu E Murtaugh MP Summerfield A. Impact of genotype 1 and 2 of porcine reproductive and respiratory syndrome viruses on interferon-alpha responses by plasmacytoid dendritic cells. Vet Res. (2013) 44:33. 10.1186/1297-9716-44-33 23675981 PMC3672080

[42] Xie J Christiaens I Yang B Breedam WV Cui T Nauwynck HJ. Molecular cloning of porcine Siglec-3, Siglec-5 and Siglec-10, and identification of Siglec-10 as an alternative receptor for porcine reproductive and respiratory syndrome virus (PRRSV). J Gen Virol. (2017) 98:2030–42. 10.1099/jgv.0.000859 28742001 PMC5656783

[43] Escalona Z Alvarez B Uenishi H Toki D Yuste M Revilla C. Molecular characterization of porcine Siglec-10 and analysis of its expression in blood and tissues. Dev Comp Immunol. (2015) 48:116–23. 10.1016/j.dci.2014.09.011 25280627

[44] Prather RS Rowland RR Ewen C Trible B Kerrigan M Bawa B An intact sialoadhesin (Sn/SIGLEC1/CD169) is not required for attachment/internalization of the porcine reproductive and respiratory syndrome virus. J Virol. (2013) 87:9538–46. 10.1128/JVI.00177-13 23785195 PMC3754101

[45] Whitworth KM Rowland RR Ewen CL Trible BR Kerrigan MA Cino-Ozuna AG. Gene-edited pigs are protected from porcine reproductive and respiratory syndrome virus. Nat Biotechnol. (2016) 34:20–2. 10.1038/nbt.3434 26641533

[46] Furuhashi K Suda T Hasegawa H Suzuki Y Hashimoto D Enomoto N. Mouse lung CD103+ and CD11bhigh dendritic cells preferentially induce distinct CD4+ T-cell responses. Am J Respir Cell Mol Biol. (2012) 46:165–72. 10.1165/rcmb.2011-0070OC 21908266

[47] Kim TS Braciale TJ. Respiratory dendritic cell subsets differ in their capacity to support the induction of virus-specific cytotoxic CD8+ T cell responses. PLoS ONE (2009) 4:e4204. 10.1371/journal.pone.0004204 19145246 PMC2615220

[48] Helft J Manicassamy B Guermonprez P Hashimoto D Silvin A Agudo J. Cross-presenting CD103+ dendritic cells are protected from influenza virus infection. J Clin Invest. (2012) 122:4037–47. 10.1172/JCI60659 23041628 PMC3484433

[49] Silvin Yu CI Lahaye X Imperatore F Brault JB Cardinaud S Becker C. Constitutive resistance to viral infection in human CD141(+) dendritic cells. Sci Immunol. (2017) 2:eaai8071. 10.1126/sciimmunol.aai8071 28783704 PMC5749640

[50] Desch AN Randolph GJ Murphy K Gautier EL Kedl RM Lahoud MH. CD103+ pulmonary dendritic cells preferentially acquire and present apoptotic cell-associated antigen. J Exp Med. (2011) 208:1789–97. 10.1084/jem.20110538 21859845 PMC3171085

[51] Heil F Hemmi H Hochrein H Ampenberger F Kirschning C Akira S. Species-specific recognition of single-stranded RNA via toll-like receptor 7 and 8. Science (2004) 303:1526–9. 10.1126/science.1093620 14976262

[52] Kono H Rock KL. How dying cells alert the immune system to danger. Nat Rev Immunol. (2008) 8:279–89. 10.1038/nri2215 18340345 PMC2763408

[53] Garcia-Nicolas O Auray G Sautter CA Rappe JC McCullough KC Ruggli N. Sensing of porcine reproductive and respiratory syndrome virus-infected macrophages by plasmacytoid dendritic cells. Front Microbiol. (2016) 7:771. 10.3389/fmicb.2016.00771 27458429 PMC4937788

