# Supplementary material for: Porcine Reproductive and Respiratory Syndrome Virus Type 1.3 Lena Triggers Conventional Dendritic Cells 1 Activation and T Helper 1 Immune Response Without Infecting Dendritic Cells
Source: Front Immunol. 2018 Oct 2;9:2299. doi: 10.3389/fimmu.2018.02299 (PMC6176214; doi:10.3389/fimmu.2018.02299)
Supplement: Supplementary file 1 [file Data_Sheet_1.PDF]

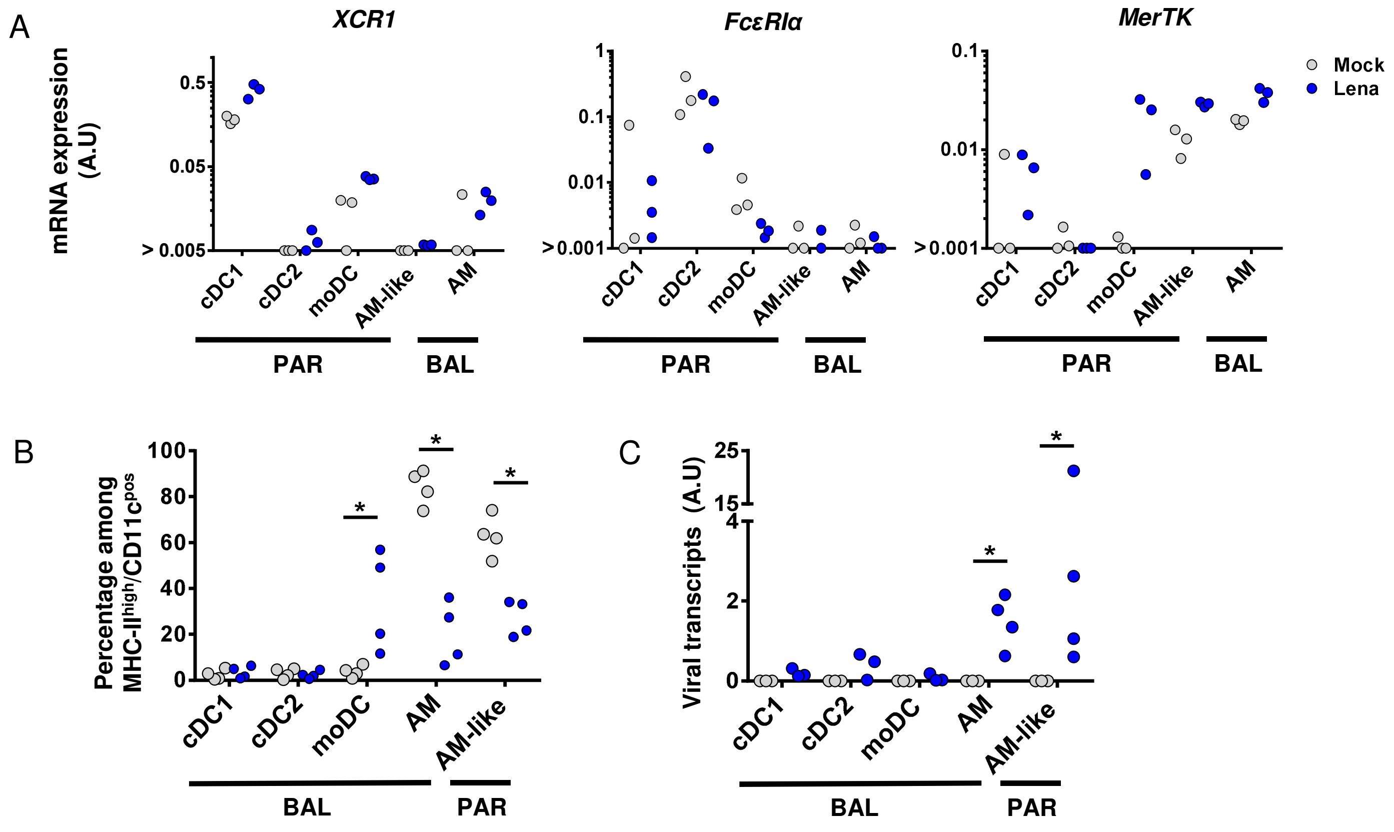

Supplementary Figure S1. Cell sorting validation, BAL DCs analysis upon Lena *in vivo* infection.
(A) MNP isolation was validated by RT-qPCR. Messenger RNA expression of *XCR1*, *FcεRIα* and *MerTK* were analyzed on control (Grey dots, Mock) and infected (Black dots, Lena) populations. Results were normalized to the reference gene *RPS24* (ribosomal protein S24) expression. Arbitrary unit: A.U. represents $2^{-\Delta Ct}$. The RT-qPCR detection threshold is at the origin of the axis. Data are representative of 3 independent cell-sorting. **(B)** Percentage of MNP were monitored in mock (Grey circles) and infected (Blue circles) broncho-alveolar lavage (BAL) after immunostaining. Data are expressed as a percentage of $MHC\text{-}II^{high}/CD11c^{pos}$ cells (see Gate1 in **Figure 1**). The Mann-Whitney's test were performed. *P-values *<0.05.*
(C) Detection of viral transcripts on sorted MNP from BAL was assessed by RT-qPCR. Primers designed on viral Nucleocapsid protein (N) were used to detect viral transcript. The Mann-Whitney's test were performed. *P-values *<0.05.*

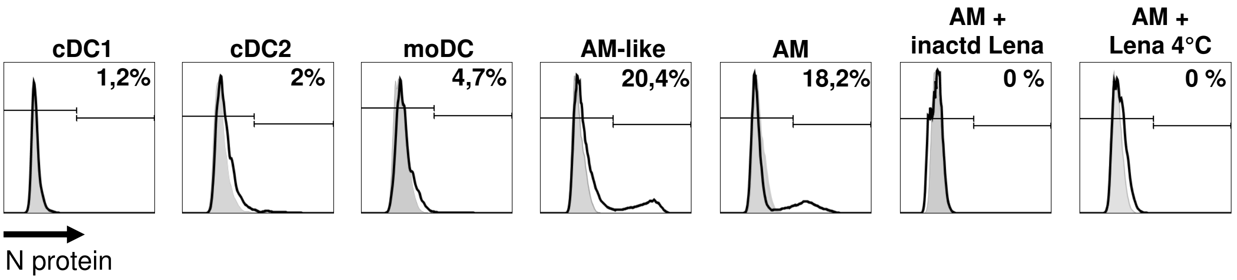

Supplementary Figure S2. *Ex vivo* Lena infection of mononuclear phagocytes using flow cytometry. Intracellular viral N protein was detected by flow cytometry in MNP-enriched parenchyma after 16 hours of Lena infection at MOI 0.5 (Open black histogram). As negative control, identical samples were incubated without virus (Closed grey histogram). Controls using inactivated (inactd) Lena and Lena incubated at 4°C with AM were performed.

A

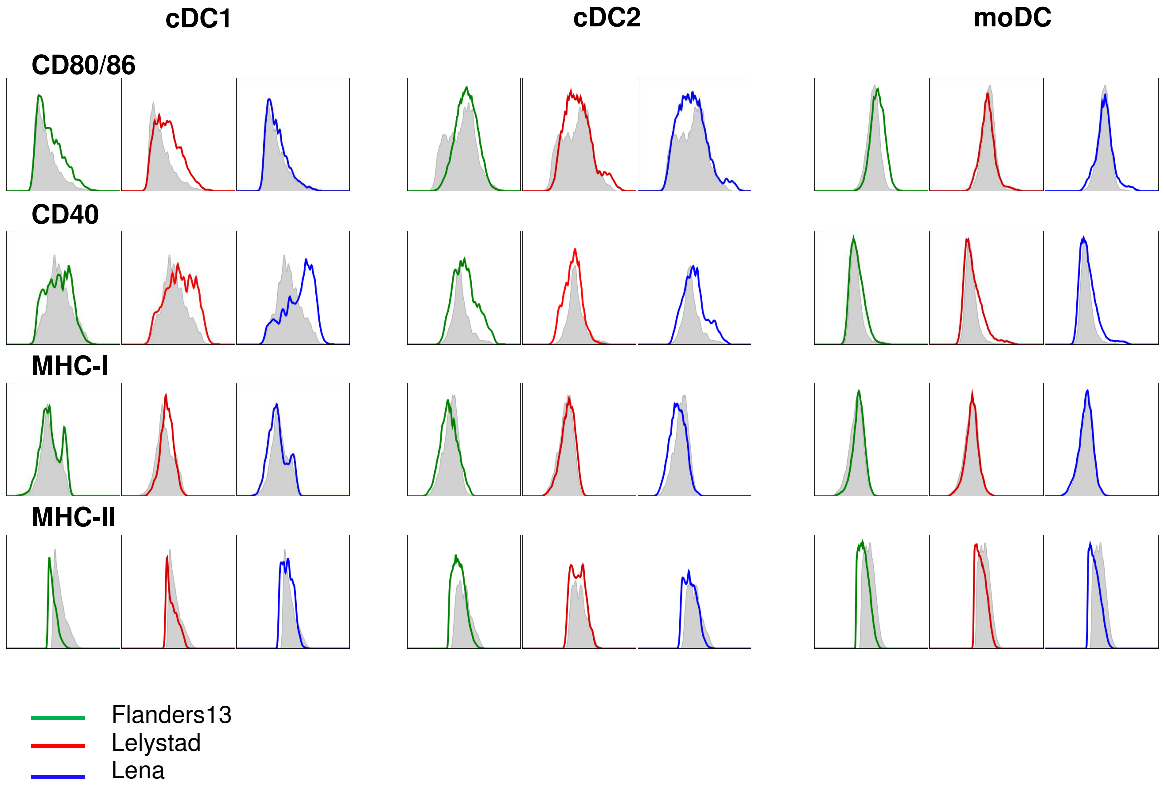

B

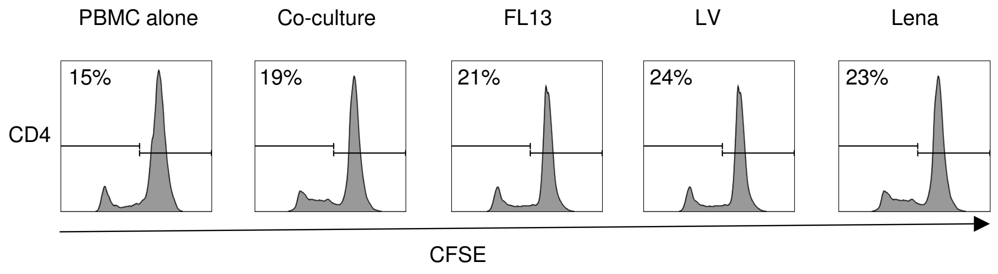

Supplementary Figure S3. PRRSV infected mononuclear phagocytes presented no difference in maturation markers expressions or allogeneic T cells proliferation induction according to virus types.

(A) Maturation markers expressions on cDC1, cDC2 and moDC relative to control (Closed grey histogram) after FL13 (Open green), LV (Open red) and Lena (Open blue) infection. Data are representative of 4 independant experiments.

(B) Percentage of proliferation of CFSE-stained $CD4^+$ after mixed lymphocytes reaction assay. Data are representative of 3 independant experiments.

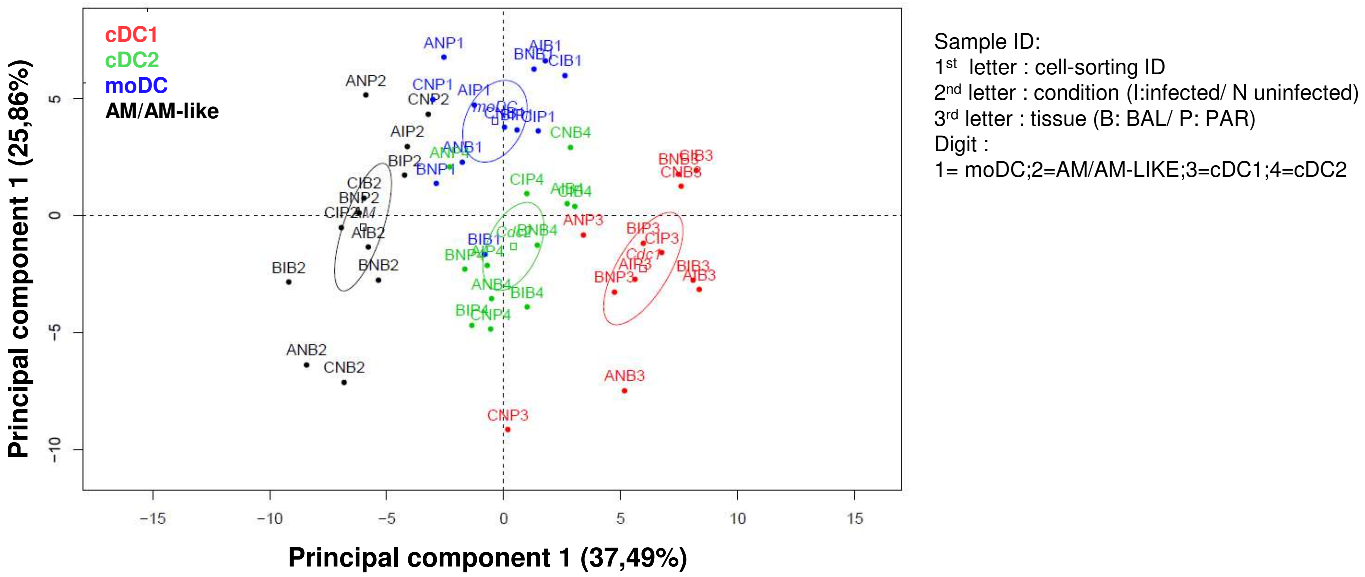

Supplementary Figure S4.

(A) Principal component analysis of cDC1, cDC2, moDC and AM/AM-like in BAL and PAR based on cytokine expression.
